# Recent Progress in Biopolymer-Based Hydrogel Materials for Biomedical Applications

**DOI:** 10.3390/ijms23031415

**Published:** 2022-01-26

**Authors:** Ayaz Mahmood, Dev Patel, Brandon Hickson, John DesRochers, Xiao Hu

**Affiliations:** 1 Department of Physics and Astronomy, Rowan University, Glassboro, NJ 08028, USA; mahmoo52@students.rowan.edu; 2 Department of Biomedical Engineering, Rowan University, Glassboro, NJ 08028, USA; pateld27@students.rowan.edu (D.P.); hickso13@students.rowan.edu (B.H.); desroc78@students.rowan.edu (J.D.); 3 Department of Molecular and Cellular Biosciences, Rowan University, Glassboro, NJ 08028, USA

**Keywords:** biopolymer, hydrogel, polysaccharide, polypeptide, protein, tissue engineering, drug delivery

## Abstract

Hydrogels from biopolymers are readily synthesized, can possess various characteristics for different applications, and have been widely used in biomedicine to help with patient treatments and outcomes. Polysaccharides, polypeptides, and nucleic acids can be produced into hydrogels, each for unique purposes depending on their qualities. Examples of polypeptide hydrogels include collagen, gelatin, and elastin, and polysaccharide hydrogels include alginate, cellulose, and glycosaminoglycan. Many different theories have been formulated to research hydrogels, which include Flory-Rehner theory, Rubber Elasticity Theory, and the calculation of porosity and pore size. All these theories take into consideration enthalpy, entropy, and other thermodynamic variables so that the structure and pore sizes of hydrogels can be formulated. Hydrogels can be fabricated in a straightforward process using a homogeneous mixture of different chemicals, depending on the intended purpose of the gel. Different types of hydrogels exist which include pH-sensitive gels, thermogels, electro-sensitive gels, and light-sensitive gels and each has its unique biomedical applications including structural capabilities, regenerative repair, or drug delivery. Major biopolymer-based hydrogels used for cell delivery include encapsulated skeletal muscle cells, osteochondral muscle cells, and stem cells being delivered to desired locations for tissue regeneration. Some examples of hydrogels used for drug and biomolecule delivery include insulin encapsulated hydrogels and hydrogels that encompass cancer drugs for desired controlled release. This review summarizes these newly developed biopolymer-based hydrogel materials that have been mainly made since 2015 and have shown to work and present more avenues for advanced medical applications.

## 1. Introduction

Biopolymers are polymers that are made up of many individual monomers, or units, within an organism. They are usually derived from plants and animals allowing them to not only be biodegradable but also easily renewable for other uses [1,2]. Biopolymers have multiple functions within the body, some of them being creating tissue made up of cells, aiding in the function of connective tissue such as human cartilage, and providing molecules to use as signals in the human endocrine system [3]. The individual monomers of the biopolymers are bonded covalently to one another allowing the polymer structure to grow and become longer. Some common examples of biopolymers include nucleic acids which make up DNA and RNA, polypeptides or proteins are made up of amino acids that are coded by nucleic acids, and polysaccharides which are complex molecules consisting of carbohydrates [4].

Polypeptides are made up of multiple different amino acids that are covalently bonded through amide bonds [5]. Polypeptides can become functional proteins after multiple polypeptides are bonded together or a single polypeptide assumes the shape needed to create a functional protein. Polypeptides are important aspects of organisms as they aid in the creation of enzymes, tissues, muscles, and are easily recognized and accepted by the immune system [6]. Polysaccharides are made up of multiple different carbohydrate units that are covalently bonded via glycosidic bonds and are important aspects of an organism as they are important for structural support of cells and organs and energy storage within the organism [4]. The single unit for polysaccharides is the monosaccharides [7]. Both polypeptides and polysaccharides can be used to create biopolymer materials that can be utilized for biomedical applications such as tissue engineering and drug delivery.

A hydrogel is a three-dimensional network of hydrophilic polymers that when in water can swell and retain the liquid. They can maintain the structure due to chemical or physical cross-linking of the polymer chains [8]. To be considered a hydrogel, the substance must constitute at least 10% of the total weight for the material [9]. There are many different theories that have been created over the years to help predict the structural outcome of hydrogels, which provides us the elasticity value of the gel, the porosity and pore size of the hydrogel network. All these theories take into consideration enthalpy, entropy, and other thermodynamic variables so that the structure and pore sizes of hydrogels can be formulated, while computational simulation methods can be further used to precisely engineer the hydrogel designed. Hydrogels can be divided into those formed by natural polymers, and those formed by synthetic polymers, and those formed by the modification of natural polymers in conjunction with synthetic linkers (called semi-synthetic hydrogels) [10]. Synthetic polymers are chemically stronger compared to natural polymers. Their mechanical strength results in very slow biodegradation rate but high durability. These two opposite properties need to be balanced through optimal design [11]. Hydrogels can be physical, chemical, or biochemical: physical gels can transition from a liquid to a gel in response to stimuli, while chemical gels rely on covalent bonding to introduce the integrity needed to form a gel; biochemical gels use agents such as enzymes or amino acids to help them through the gel process [12]. Depending on the ionic charges on the bound groups, the gels may be cationic, anionic, or neutral [9]. Therefore, hydrogels can undergo conformational transitions, in response to a stimulus, this can include chemical or physical stimuli. Shape-changing hydrogels can be created by stacking different hydrogel layers that each react to a different stimulus [13]. Based on the different methods of polymerization such as suspension, block, solution, and emulsion, hydrogels may also be classified as homopolymer or copolymer [9,14]. Homopolymers only contain one type of monomer in their structure and based on the nature of the monomer and the technique used for polymerization, they may have a cross-linked structure. Copolymeric hydrogels are composed of two types of monomers, of which at least one is hydrophilic. By combining two polymers, an interpenetrating network (IPN) can be created, provided that one of them is already present in the solution [9,14].

Hydrogels are used in many fields, and this is due to their specific structures and compatibility with different conditions of use [3,14]. Flexibility is what distinguishes hydrogels from other biomaterials, and its versatility is unmatched as its uses can range from industrial applications to biological applications. Major applications of hydrogels include drug delivery, dye and heavy metal removal, scaffolds in tissue engineering, and even contact lenses [3,9,14]. Biopolymers that were introduced earlier are key to producing useful and innovative hydrogels with better biocompatibility [15]. Both sugar-based polysaccharides biopolymers, and protein-based polypeptides, have significant use in the creation of new biodegradable and biocompatible hydrogel compounds. Specific examples of polysaccharides used in hydrogel construction include cellulose, alginate, and glycosaminoglycans. For polypeptides, this may include collagen, gelatin, and elastin [16]. These are the major ingredients in the creation of hydrogel, and each has their own specific benefits and structural capabilities creating a variety of hydrogels that can be tuned to any need. Due to these specific properties, these hydrogels can effectively help deliver cells and molecules to desired locations with human bodies. Other hydrogels composed from polypeptides and polysaccharides are able to deliver cancer drugs to desired locations to prevent the spread of cancer, regenerate tissue that may have been damaged due to illness or injury, and delivery molecules such as insulin for diabetic patients. A specific example would be a alginate-gum tragacanth hydrogel, a hydrogel composed of a polysaccharide, which released insulin in certain pH levels leading to release of insulin in only desired areas [17]. Additionally, nucleic acids, such as DNA, can be formulated into hydrogels for other biomedical applications [18]. These examples will be discussed in further detail later in the review, to gain some perspective on the hydrogel opportunities that biopolymers have in the field.

In this review, we will define hydrogels, present possible fabrication methods, and show their different applications, specifically in the biomedical field. Different hydrogels will be discussed, including those constructed from both polypeptide and polysaccharide biopolymers. Fabrications methods for these hydrogels will be described as well, Fabrication methods that will be reviewed include a batch reactor chamber, 3D printing, physical cross linking, and topological entanglement. These methods all help create biopolymer-based hydrogels that can later be used for major biomedical applications [19]. Lastly, some of the biomedical applications of these hydrogels as well as clinic data associated with each application will be discussed. The biomedical applications include cancer therapeutics, self-healing applications, drug delivery, and neural, skeletal, and cardiovascular regeneration.

## 2. Biopolymer Materials

### 2.1. Polypeptides

**Figure 1:**
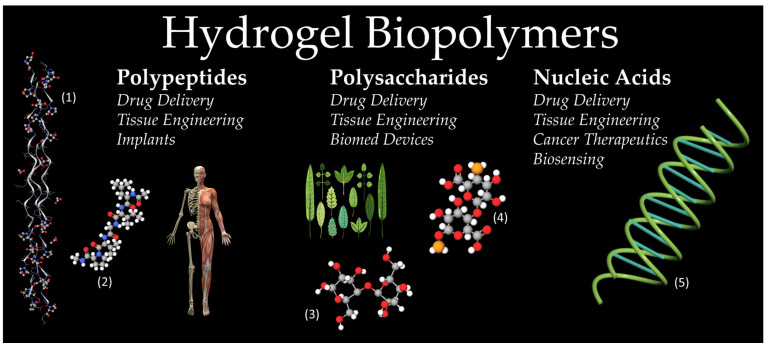
Examples of typical hydrogel biopolymers. Polypeptide column showcases collagen (**1**) and elastin (**2**) structures, which are found in musculoskeletal systems. Polysaccharide column shows the cellulose (**3**) structure and alginate (**4**) structures, as the majority of polysaccharides are derived from plant life. A double-helix DNA molecule (**5**) is shown as a typical nucleic acid. (Images of the musculoskeletal system and the plant life are open source provided by pixabay.com, and images of collagen, elastin, cellulose and alginate are open source provided by molview.org).

Polypeptides, more commonly referred to as proteins, are made up of multiple different amino acids that are covalently bonded via amide bonds (Figure 1). Polypeptides can then become functional proteins after multiple polypeptides are bonded together or a single polypeptide assumes the shape needed to create a functional protein. Polypeptides are important aspects of organisms as they aid in the creation of enzymes, tissues, and muscles [20]. Based on the conformation of their amino acid chain, proteins are placed into one of four levels of structure: primary, secondary, tertiary, or quaternary [5,20]. The primary structure is the sequence of amino acids in and of itself. The secondary structure introduces some details regarding the orientation of the chain. Helices, sheets, loops, and turns, are all aspects that make up the secondary structure of a protein [5,20]. The tertiary structure includes domains, folds, and motifs, organizational terms used to describe unique regions of the amino acid chain that follow a similar trend [20]. Lastly, quaternary structure, which isn’t necessarily reached in all functional proteins, is the intertwining of multiple separate polypeptide chains to create a new functional protein created via the arrangement of these multiple chains [5,20].

#### 2.1.1. Collagen

Collagen is one of the primary proteins that make up the extracellular matrix (ECM) [21]. Its primary role in the extracellular matrix is to provide structural support, a feature that makes said protein an excellent tool for scaffolding and hydrogel application. Collagen is a trimeric molecule composed of three, intertwined, alpha-helices [22]. The ubiquity of hydrogen bonding within the triple helix structure is what gives collagen its immense tensile strength. It is also interesting to note that while a certain amount of crosslinking is good for collagens structure, an excessive amount leads to an increasingly brittle structure, a common symptom of aging [23]. Buehler et al. studied the mechanical properties of collagen fibrils and suggested that nature has refined a length for the tropocollagen (TC) monomers that maximizes the strength of assembled collagen fibrils. Simulations indicated that TC monomers either longer or shorter than ~300 nm would form collagen fibrils with less favorable mechanical properties [23]. An example of collagen used as a hydrogel are collagen-phosphorylcholine hydrogels that have been demonstrated in the application of regenerative medicine [24].

#### 2.1.2. Gelatin

Gelatin is derived from collagen from different sources, and the manufacturing process from collagen influences gelatin properties, including its molecular weight and isoelectric point [25]. Gelatin extraction from collagen requires a pre-treatment to cleave the cross-links that stabilize the collagen structure, these treatments can be based on basic, acidic, or an enzymatic process. The most common are the base and acid treatments as acid treatment results in gelatin with isoelectric point 8–9 (“type A” gelatin), while base treatment results in gelatin with isoelectric point 4–5 (“type B” gelatin) [26,27]. When extracting the gelatin, the degree of collagen converted into gelatin can be optimized by temperature, pH, and processing time [21,24,28]. Gelatin hydrogels have demonstrated self-healing capability and are advantageous in their biocompatibility and biodegradability and have been used in injections for therapeutic purposes [28]. The reason gelatin has been sought out as a means for hydrogel applications is that it is a solution to the disadvantages of using its parent compound, collagen. For example, despite collagen being useful as a biomaterial due to its unique properties, issues lie with adverse immunological responses and lack of mechanical and thermal stability without additional cross-linking compounds [29]. To provide an example, collagen can be extracted from fish scales into gelatin to yield better biocompatibility, degradability, and lower cytotoxicity, especially when combined with different phenol acids [29].

#### 2.1.3. Elastin

Elastin is one of the most significant proteins found in the extracellular matrix [3,21,30]. As the name suggests, the focus of elastin is its elastic recoiling ability. Regions of the human body that this is especially useful include the aorta, bladder, skin, and lung [30]. Tropoelastin is the precursor to elastin and the elastin gene is expressed increasingly in areas of injury [31]. In addition to its recoil and resilience, elastin is chemically inert, increasing its biocompatibility, making it a prime candidate for hydrogel applications [30]. Elastin was used to develop hydrogels that were crosslinked and highly porous. The elastin’s resilience allowed a hydrogel to have high porosity to be constructed successfully, resulting in a scaffold that encouraged high levels of cell penetration [32]. This elastin hydrogel specifically showed the great potential elastin has for applications within tissue regeneration [32].

### 2.2. Polysaccharides

Polysaccharides are made up of multiple different carbohydrate units that are covalently bonded via glycosidic bonds (Figure 1). The single unit for polysaccharides is the monosaccharides [33]. Polysaccharides are important aspects of an organism as they are important for the structural support of cells and organs and energy storage within the organism [34]. The structural category of polysaccharides includes cellulose and chitin, whereas saccharides used for energy, or storage, include starch and glycogen [35,36]. Polysaccharides can be successful as hydrogel materials due to their low cytotoxicity and high stability, often being more stable than proteins since they are not denatured with high temperatures [36].

#### 2.2.1. Alginate

Most frequently extracted from brown seaweed (Phaeophyceae), alginate is a polysaccharide with desirable traits for hydrogel applications, such as its favorable biocompatibility and gelation capability [37]. The brown seaweed is treated and filtered with NaOH and attached to calcium chloride, precipitating the resultant alginate. Alginate is typically formed with the heavy metal ion absorption technique that strengths alginate hydrogels much more than a non-absorbed version and makes it a better hydrogel overall for recovery [38]. Over 200 different forms of alginates can be produced and sold, and this has to do with their slight chemical differences, specifically their M and G residue ratio of copolymer blocks. The M residue is (1,4)-linked-Beta d-mannuronate and G are Alpha-l-guluronate [37]. This ratio can also be tweaked to achieve the most ideal polymer for the application at hand, for example, some have the most biocompatibility [37]. One case of alginate hydrogel formation for example is alginate hydrogels formed in the shape of beads and fibers for cell delivery within the field of tissue engineering [39,40].

#### 2.2.2. Cellulose

Cellulose is the most frequently found polysaccharide on the entire globe [41,42]. It is primarily seen in nature as a structural polymer in plant life. Cellulose is derived from glucose, and its chains are connected through Beta-1-4-glycosidic bonds. Cellulase is the enzyme capable of hydrolyzing these bonds and thus breaking down cellulose [42]. The structural function along with good biocompatibility of cellulose molecules make them an excellent contender for hydrogel use, especially for drug delivery. The inclusion of cellulose and its derivatives in hydrogels leads to structural and morphological changes in hydrogel system in terms of enhancement of pore sizes due to repulsive forces of intra carboxyl groups directing to a large swelling ratio [43,44,45]. Thus, cellulose incorporation in hydrogels makes them an appropriate candidate for a manageable drug release. Additionally, since they are found innumerably in nature, they are very easy to extract for use since there are so many potential sources. For example, cellulose has been used in the form of green bacterial cellulose to develop new hydrogel materials. Some sources for derivation include isolation from ripe fruits along with a liquid potato medium [46].

#### 2.2.3. Glycosaminoglycans

In addition to polypeptides and polysaccharides being capable of forming hydrogels, a mixture of these molecules can also be used to formulate new hydrogel compounds. The extracellular matrix (ECM) is composed of both fibrous proteins and a special polysaccharide compound called glycosaminoglycans (GAGs) [47]. Glycosaminoglycans are a linear chain of repeating disaccharide units and are important in the ECM as they provide a scaffold for cell adhesion and proliferation. Decellularized GAG-ECM composite hydrogels have been constructed to form a hydrogel with the ability to have a solid scaffold from the ECM in addition to adjustable gelation kinetics [47].

### 2.3. Nucleic Acids

Nucleic acids are unique macromolecules and are found in all cells and viruses [48,49]. The two most discussed nucleic acids are deoxyribonucleic acid (DNA) and ribonucleic acid (RNA) (Figure 1). The molecules are composed of a chain of ribose and phosphate units, with a nitrogenous base attached to each ribose. The nitrogenous bases are split into purines, double-ringed molecules, that include adenine and guanine, and pyrimidines, single-ringed molecules including cytosine, thymine for DNA, and Uracil for RNA [49,50]. DNA is a double helix, whereas RNA is single-stranded [49]. The function of DNA is to encode the instructions for the cell to produce various proteins. RNA has various forms, such as mRNA, tRNA, and rRNA, all of which work together with the DNA to synthesize proteins inside the cell [48,49]. Recently, these molecules and their unique properties have been researched in their potential applications as hydrogel materials, specifically in the fields of drug delivery, tissue engineering, cancer therapeutics, and biosensing [18,49].

DNA hydrogels constructed via crosslinking are a prime tool for drug delivery due to their porosity, biocompatibility, and ability to program the DNA sequence to one’s liking [51]. DNA-based hydrogels utilized for biosensing are an effective solution since they are an affordable, programmable, and sensitive platform for biosensing. One such design for these biosensors includes using a synthetically created polymer as a scaffold, and a functional DNA cross-linker as a sensor [52]. Once the DNA sensor recognizes the target compound, the appearance can be reported through a variety of mechanisms [51,53]. The hydrogel might grow in volume or mass, or there can be a readable signal, such as a fluorescent response [53]. DNA hydrogels’ capability to biosense, scaffold, and drug deliver makes them a prime candidate for novel cancer therapeutics [18,53].

## 3. Gel Theory and Structure

### 3.1. Gel Theory

Hydrogels are polymeric materials with a structure that has hydrophilic polymer chains and can retain a large volume of water in their interstitial structures [8]. When in contact with water hydrogels can continue to swell and absorb it due to the presence of hydrophilic groups, and this type of crosslinking helps prevent hydrogels from dissolving [8]. Multiple gelation theories can be used for biopolymer-based hydrogels include Flory-Rehner’s theory and Peppas’s theories [54,55,56,57,58,59,60].

#### 3.1.1. Flory-Rehner Theory

Flory-Rehner’s equation describes the mixing of polymers and liquid molecules, and these can be used to analyze hydrogels without having to worry about ionic domains [54]. These domains actively release ions as the hydrogel deforms and this is used to help study with analyzing the structure of the gel. The theory gives the change of free energy when the polymer increases in size and is described with Equation (1) below,

(1) $$\Delta G_{total}=\Delta G_{elastic}+\Delta G_{mixing}$$

where ∆*G_elastic_* comes from the elastic stored forces in the polymer chains contained in the gel networks; ∆*G_mixing_* is the result of the mixing between fluid molecules with the polymer chains. The mixing factor measures the compatibility between the solvent molecules and the polymer. To find the equilibrium status, we can differentiate this to Equation (2),

(2) $$\mu_{1}-\mu_{1,0}=\Delta \mu_{elastic}+\Delta \mu_{mixing}$$

where the chemical potential of the inside of the gel and outside of the gel must be equal, which means that the elastic and mixing forces must cancel each other out. There are multiple derivations for the equation, but in its full form, the Flory-Rehner Equation (3) is down below,

(3) $$-\left[\ln\left(1-v_{2}\right)+v_{2}+v_{2}^{2}\right]=\frac{V_{1}}{\bar{v}M_{c}}\left(1-\frac{2M_{c}}{M}\right)\left(v_{2}^{\frac{1}{3}}-\frac{v_{2}}{2}\right)$$

where $v_{2}$ is the volume fraction of polymer in the swollen mass, $V_{1}$ the molar volume of the solvent, $\chi_{1}$ is the Flory solver-polymer interaction term, $\bar{v}$ is the specific volume of the polymer, M is the primary molecule mass, and $M_{c}$ is the average molecular mass between crosslinks [55,56].

Therefore, Flory-Rehner’s equation considers three main ideas. First, entropy changes occur when mixing the polymer and the solvent, and in gels, this entropy change favors swelling because entropy is positive. Second, this change is caused by the reduction of possible conformations as the networks swell, and this change is negative if the swelling decreases. The third is the heat when mixing a polymer and solvent where usually is positive and that it opposes mixing [55].

#### 3.1.2. Rubber Elasticity Theory

Hydrogels resemble natural rubbers that deform with stress due to their elastic nature. Flory has previously used the elastic properties of hydrogels to analyze and describe the structure [55]. The issue is that the Flory-Rehner theory cannot be used to analyze the elasticity of hydrogels in a solvent, and this is where the Peppas-Theory comes in, as the Equation (4) can be used to analyze hydrogels prepared in a solvent [8,57].

(4) $$\tau =\frac{\varrho RT}{\bar{M_{c}}}\left(1-\frac{2\bar{M_{c}}}{\bar{M_{n}}}\right)\left(\alpha -\frac{1}{\alpha^{2}}\right){\left(\frac{v_{2,s}}{v_{2,x}}\right)}^{\frac{1}{3}}\;\;$$

where *τ* is the applied stress to the polymer sample, *ρ* is the density of the polymer, α is elongation ratio and *M*_c_ is the molecular weight between crosslinks. Experiments must be done in tensile mode, or mechanical stress must be put on to analyze the hydrogel using this theory [58].

#### 3.1.3. Calculation of Pore Size

The pore size of a hydrogel is responsible for its diffusion properties. Depending on the size of the pores this can be used to selectively decide how drug delivery or other mechanisms will interact with the gel [59]. The size of the pore is described by a structural parameter such as the linear distance between two neighboring cross-links [59]. We can then use equations and substitute them to find the correlated distance of polymer chains. By combining the correlation length, elongation ratio, the end-to-end distance of the polymer chain, length of the bond along the polymer backbone, and the molecular weight of the units, we are led to the Equation (5) below. This can be used to calculate the correlated distance of the polymer chains between two adjacent crosslinking points,

(5) $$\varepsilon =v_{2,s}^{-\frac{1}{3}}{\left(\frac{2C_{n}\bar{M_{c}}}{M_{r}}\right)}^{\frac{1}{2}}$$

where ε is the correlation length, $v_{2,s}$ is the volume fraction of the swollen polymer, $C_{n}$ is the Flory characteristic ratio, *M_r_* is the molecular weight of the repeat unit, and *M_c_* is the molecular weight between crosslinks [60]. In addition, this equation can be also used for computational methods to predict the structure that can be formed depending on the materials used and the type of hydrogel we are making [60].

### 3.2. Gel Structure

The mechanical properties of hydrogel are dependent on the interior structure, and there are many ingenious ways to obtain desired mechanical properties from a hydrogel by manipulating its network composition and density. A good example is double-network hydrogels (DN Gels), as they utilize two different network compositions to create a hydrogel that can resist shear stress and tension forces. The first network uses a dense network component to resist the shear forces, while the second network has hidden polymer chains that can elongate to withstand tension forces and resist deformation [61]. Many biomaterials are tunable, meaning that we can engineer the network composition to our desire and create innovative hydrogels with varying properties for each specific need. The previously mentioned theories can be used with computational methods to then expedite the process by being able to efficiently predict what is needed to create the desired network and structure for the application at hand.

### 3.3. Different Types of Hydrogels

There are many different biopolymer-based hydrogels, but the main ones include pH-sensitive gels, temperature-sensitive gels, electrosensitive gels, and light-responsive gels [62,63,64,65,66,67,68,69]. Table 1 shows the different types of hydrogels, the stimulus that they respond to, and the potential applications that they have in the biomedical field.

#### 3.3.1. pH-Sensitive Hydrogels

Any pH-sensitive polymer must contain a hanging acidic or basic group that responds to an environmental change in pH occurs [62]. Amphiphilic hydrogels contain both acidic and basic groups which can then exhibit a two-phase transition in both basic and acidic environments. The two phases include a separated, gel-like phase that is formed by polymer-polymer interactions, and while in this phase maximum hydrophobicity takes place which causes a maximum shrinkage of the hydrogel. The second phase has interactions between the solvent and the polymer and creates a mixed phase in which the polymer and solvent are mixed and produce the maximal value of hydrophilicity [63].

#### 3.3.2. Thermogels

Thermo-gels are hydrogels that are temperature sensitive and can form a gel when there is a temperature change. These gels contain primarily methyl, ethyl, and propyl groups which interact with water molecules by hydrogen bonds and cause the gel to swell [64]. These bonds are correlated with the temperature. The main characteristic of these gels is the presence of the hydrophobic groups [64]. Most polymers increase water solubility as the temperature increases. However, there are cases where the inverse happens, and this behavior causes a polymer phase transition as the temperature is raised to a critical value called “lower critical solution temperature” (LCST) [64]. As the temperature increases, positive thermosensitive hydrogels exhibit the opposite behavior of negative thermosensitive hydrogels. LCST can be increased by adding a hydrophilic component which makes it a very good candidate for drug delivery. poly(*N*-isopropylacrylamide) (PNIPAM) is the most studied thermosensitive hydrogel in tissue engineering investigations. Other examples of thermosensitive hydrogels are collagen, agarose, hyaluronic acid, poly(organophosphazenes), and chitosan [2,65]. Thermogels are also useful for lowering the critical micelle concentration (CMC), which means that micelles for drug delivery are much more easily formed than typical hydrogels and offer greater stability. This leads to a higher percentage of micelles reaching the targeted area for drug delivery and can provide better outcomes in medical usage [70].

#### 3.3.3. Electro-Sensitive Hydrogels

Electrosensitive hydrogels undergo shrinkage or swelling based on the presence of an electric field [66,67]. Like pH-sensitive hydrogels, they are comprised of polyelectrolytes. Under electric fields counterions and immobile charged groups are produced in the network and the result is that the hydrogel can shrink and swell regionally at the anode and cathode. This leads to bending of the hydrogel, and that bending depends on the structure of the hydrogel, strength and direction of the electric field, and duration of its application [66,67]. Hydrogels with acrylamide and carboxylic acid derivatives have been utilized as electro-sensitive and biocompatible smart muscle-based devices [68]. Benefits of such hydrogels include applications in sound dampening, chemical separations, and controlled drug delivery [68]. Therefore, electrosensitive hydrogels are gaining an increase in popularity as they can be used in biosensors and functional tissue-engineered scaffolds and implants. In combination with 3D printing technology, this class of hydrogel materials might be one of the most advanced approaches towards future biomedical applications [71].

#### 3.3.4. Light Sensitive Gels

Light-sensitive gels can change their properties when a certain wavelength is passed through [69]. These changes are typically done with chromophores, which are light-responsive functional groups, and these changes can include expansion or shrinkage of the gel. It is important to note that light-responsive hydrogels are also susceptible to pH changes [69]. One exception to having chromophores as a functional group to change gel structure is to have functional groups that can also be heat sensitive, that can be radiated by a certain wavelength to cause heat changes [69].

#### 3.3.5. Shape-Changing Gels

A shape-changing hydrogel can change its shape when a certain external stimulus, such as pH change, temperature change, or addition of aqueous solutes act upon the gel. These changes occur due to the differences in swellings within different regions or zones of the gel. These shape-changing gels may reverse their shape if the pH or temperature is restored to its original state. Many shape-changing gels are originally in the form of flat films that are self-folded or rolled into particular shapes such as tubes [13]. These can be incredibly useful for cell implantations. For example, during the implantation of bone marrow cells, the hydrogel can be turned into a tubular shape once inside the bone to ensure maximum surface area contact between the hydrogel and the bone, resulting in more cell proliferation and leading to faster cell delivery [13]. Having these hydrogels with changing shapes is an incredible innovation for the future of cell delivery, as their versatility makes them excellent candidates for such applications.

## 4. Fabrication Methods

### 4.1. Creating Hydrogels

**Figure 2:**
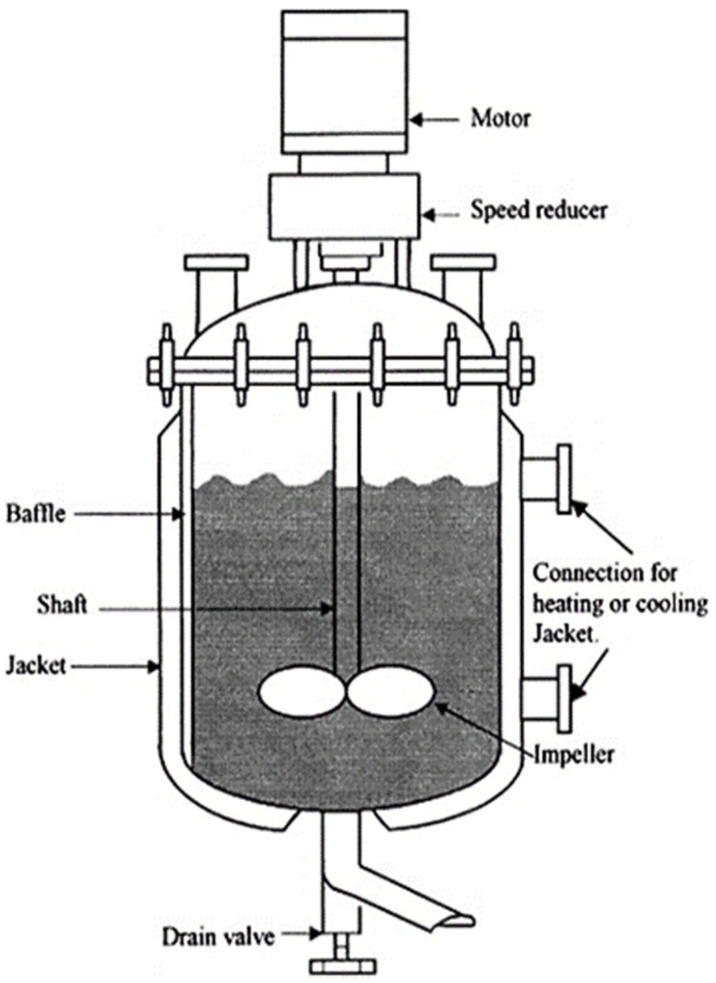
Hydrogel fabrication method in controlled batch reactor chamber. Parts such as motor, speed reducer, baffle, shaft, jacket, connection for heating or cooling, impeller, and drain valve are presented [19]. Adapted with permission from Elsevier, 2015.

Hydrogels are largely fabricated using a variety of materials from several suppliers [72]. For example, when creating an injectable high-strength hydrogel, one study used Ethylene oxide, potassium, naphthalene, diphenyl methane, pentaerythritol, anhydrous dimethylsulfoxide (DMSO), and several other compounds to synthesize their gel [72]. These reactants are used to formulate a hydrogel by inserting all the chemicals into a reactor that mixes everything at a specified stir rate and specified heat (Figure 2) [19]. After these chemicals form a solution, this solution is mixed with other pre-prepared solutions to create a hydrogel with the high strength properties needed to withstand the large forces in cartilage tissue.

### 4.2. 3-D Printing Hydrogels

**Figure 3:**
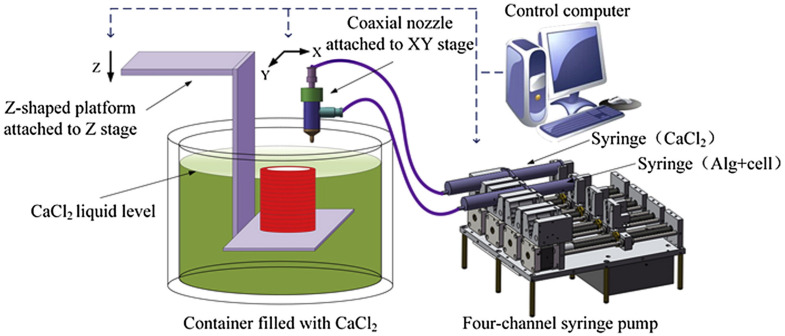
3D printing/bioprinting system with a coaxial nozzle. This is an example of alginate and cells (first syringe) being printed with CaCl_2_ (second syringe) on a Z-shaped platform from the coaxial nozzle in a CaCl_2_ liquid bath [74]. Adapted with permission from Elsevier, 2015.

Bio-applications for 3D printing have started to revolutionize the industry of biomaterials. Biodegradable materials have been applied to biological settings to create extracellular microenvironments, vasculature networks, and more. However, hydrogels have benefitted greatly from bio-printing with 3D printed scaffolds. The hydrogel can swell in water and is polymer-based. These polymers can be formulated to crosslink in a way that provides a flexible, yet rigid support structure for other cellular processes [73]. Hydrogels can be tailored for specific purposes and use depending on the type of material and print that is associated with a particular task (Figure 3) [74].

### 4.3. Physical Crosslinking

**Figure 4:**
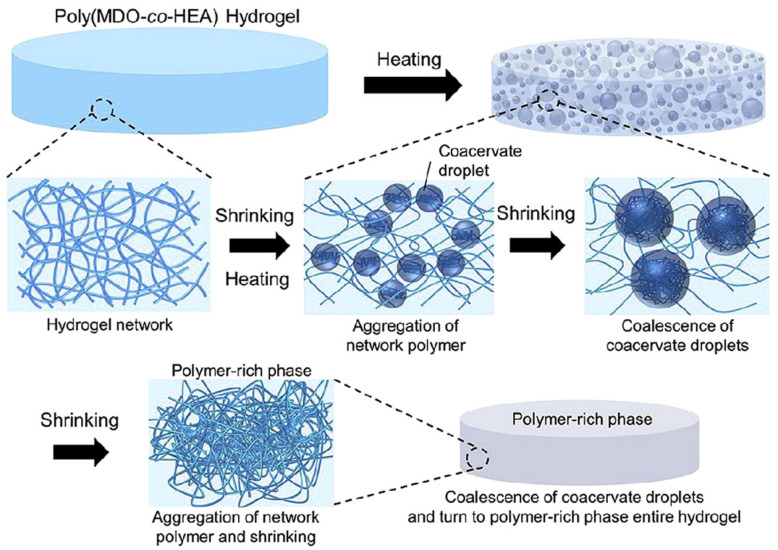
Hydrogel crosslinking on the molecular level, displaying change from raw material and solution to finished crosslinked hydrogel product that can be later used for several different biomedical applications [77]. Adapted with permission from Elsevier, 2019.

Crosslinking can be used as a micro polymeric connection within the gel. Depending on how the gel is created, this can either strengthen or weaken the gel [75]. While crosslinking can be used for the physical characterization of the gel, it can also be used as a method of making the gel unique. Crosslinking can signal reactive sites and incorporate signals and cues for where the gel should bond to different points in the injection site for a more adhesive fit, drug release, etc. [76]. This process can be conducted in several ways, both physical and chemical (Figure 4) [77]. Physical methods include imine bonding, motel-complexation, and disulfide bonds to create strong structures through chemical means. Physical alternatives include hydrogen bonds and host-guest interactions [76,77].

### 4.4. Topological Entanglement

Topological entanglement occurs in hydrogels when chains of polymers are extended using thiol chemistry [78]. The extension chemicals have a secondary purpose of binding with one another, creating a polymeric network within the hydrogel. This only enhances the many applications of hydrogels including tissue adhesion, wound closure, and drug delivery [79]. Chitosan networks are the main component of topological entanglement, resulting in strong yet flexible networks for hydrogels to carry out their intended function. The formation of these networks in a localized portion of the hydrogel is confirmed by fluorescent labeling of the chitosan and tracking their diffusion throughout an area with a microscope [79].

## 5. Recent Applications

### 5.1. Cell Delivery for Tissue Regeneration

Due to their incredible biocompatibility, facile methods of synthesis, and range of materials able to be used [81], biopolymer-based hydrogels have recently been used as cell delivery methods for different tissue regeneration such as skeletal muscle tissue, osteochondral tissue, and wound healing (Table 2). These hydrogels contain cells for specified regions of the body that may be released from the hydrogel to aid in tissue regeneration and cell proliferation. Some hydrogels encapsulate stem cells allowing these cells to differentiate into cells for specific tissues. Recently, injectable hydrogels have been found to have tremendous potential in use as cell scaffolds for tissue engineering applications [73]. Using hydrogels, tissues such as skeletal muscles, cardiac tissues, and areas wounded can be treated and repaired without major invasive procedures such as surgery [76].

#### 5.1.1. Skeletal Muscle Regeneration

**Figure 5:**
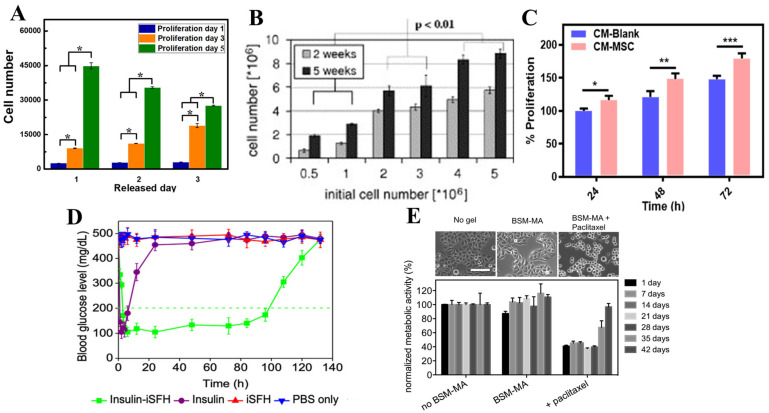
(**A**) The proliferation of the C2C12 cells on dextran-graft-aniline tetramer-graft-4-formylbenzoic acid and N-carboxyethyl chitosan hydrogels after being released for 1–3 days (* *p* < 0.05) [82]. (**B**) The effects of the initial chondrocyte cell number on the cell proliferation of fibrin with calcium ion hydrogels in weeks 2 and 5 [85]. (**C**) The proliferation of fibroblast from carboxymethyl chitosan and carboxymethyl cellulose dialdehyde hydrogel with and without mesenchymal stem cells (*** *p* ≤ 0.001, ** *p* ≤ 0.01, and * *p* ≤ 0.05) [86]. (**D**) Blood glucose levels with several methods demonstrate that insulin-loaded injectable silk-fibroin protein hydrogel maintains blood glucose levels the longest [87]. (**E**) Changes in morphological and metabolic activity of HeLA epithelial cancer cells during release of anti-cancer drug paclitaxel from mucin glycoprotein-based hydrogel (scale bar is 40 μm) [88]. Reproduced with permissions: (**A**) is from Elsevier, 2019; (**B**) is from Elsevier, 2007; (**C**) is from American Chemical Society, 2019; (**D**) is from American Chemical Society, 2020; (**E**) is from Elsevier, 2015.

Hydrogels used to help with the regeneration of skeletal muscles are often self-healing hydrogels that work to release skeletal muscle cells that aid in regeneration. These regenerated muscles are durable and flexible and can act as natural muscles when being used in intense physical situations. An example of a biocompatible self-healing skeletal hydrogel was one made from dextran-graft-aniline tetramer-graft-4-formylbenzoic acid and N-carboxyethyl chitosan. This hydrogel contained C2C12 cells that were released from the hydrogel at a linear rate. This hydrogel containing the C2C12 cells helped with muscle regeneration as they were still able to proliferate and promote skeletal muscle regeneration as seen in Figure 5A [76,82]. Another major example of cell delivery for skeletal muscle regeneration was the hyaluronic acid-chitosan (HA-CS) hydrogel system that encapsulated C2C12 cells as well [80]. This hydrogel system aided in the regeneration of myofibers and was able to help with blood vessels and nerve formations in the four weeks that it was implanted into mice [80].

The excellent biocompatibility of gelatin methacryloyl (GelMA) hydrogels make them suitable as cell culture matrices that mimic native ECM. However, to fabricate tissue constructs like living tissues, one of the essential requirements is to generate organized assemblies of various types of cells to resemble the complex architectures of the targeting tissues in vitro [83]. A hydrogel composed of GelMA and poly(3,4-ethylenedioxythiophene) nanoparticles (PEDOT NPs) also encapsulated C2C12 cells for the proliferation of muscle fibers [84]. This hydrogel was able to help proliferate muscle tissue and was shown to proliferate better than hydrogels composed of just Gelatin methacryloyl [84]. These C2C12 encapsulated hydrogels demonstrate the practical uses of hydrogels to help aid in skeletal muscle regeneration allowing for a better method to regain muscle from volumetric muscle loss.

#### 5.1.2. Osteochondral Tissue Regeneration

Hydrogels used for the regeneration of osteochondral tissue are often self-healing constructs used to aid with cell proliferation and regeneration. Using hydrogels for osteochondral tissue is very important as the cartilage between bones is very avascular but prevents bones from grinding each other causing bone deformation [72]. An example of biopolymer hydrogels is an agarose/silk fibroin blended hydrogel that had good viability for the seeded chondrocytes. The in vitro testing of this agarose/silk fibroin hydrogel demonstrated proliferation of the chondrocytes, higher stability for load bearing due to the addition of silk fibers, and more increased collagen production [89]. Another example of hydrogels used for regeneration of osteochondral tissue is microfiber-reinforced silk hydrogels that were seeded with chondrocytes. Results from the testing of this hydrogel demonstrated creation of cartilage constructs that had a similar equilibrium modulus to cartilage made in the body [90]. A hydrogel made of fibrin gels with calcium ions containing suspended bovine chondrocytes also demonstrated proliferation. In this experiment, extracellular matrices were being formed as cells were proliferating as seen in Figure 5B [85]. This demonstrated the potential use of this hydrogel matrix to be a possible solution to osteochondral regeneration for cartilage damage [85]. These hydrogels present a future avenue for osteochondral regeneration without heavy invasive intervention.

Skull tissues is much harder to regenerate than other bones in the body, and Tabata et al. investigated how hydrogels can be used to regenerate skull deficits [91]. The extent of bone regeneration induced by gelatin hydrogels incorporating 100 lg of bFGF increased with a decrease in their water content. Further examination showed that more slowly degrading hydrogels of lower water content prolonged the retention period of osteoblasts in the bone defects. This led to enhanced bone regeneration compared with other hydrogels with higher water content [91]. Bone regeneration is one of the biggest areas where hydrogels can be used prominently, and it is believed that in the future this will be a major player in osteochondral related diseases and illnesses. From the above studies, it can be concluded that biopolymer-based hydrogels that are tunable with their compositions are more advantageous for osteochondral biomedical applications.

#### 5.1.3. Wound Repair and Regeneration

**Figure 6:**
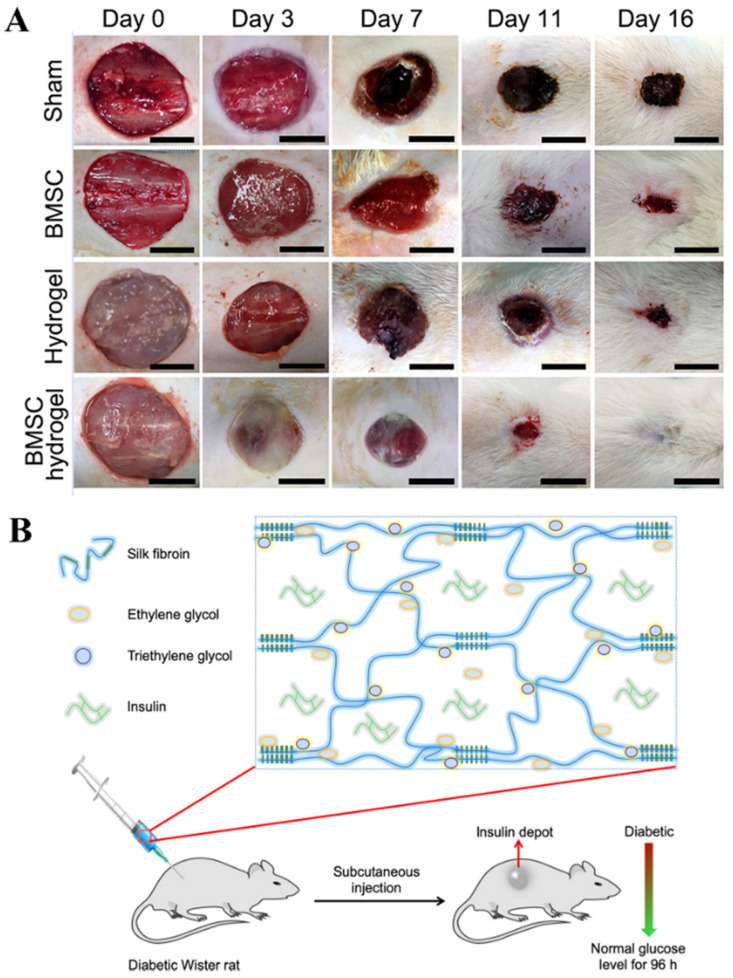
(**A**) Wound closure after different treatments visually presenting that chitin nanofiber-based hydrogel encapsulating bone marrow mesenchymal cells provide the best outcomes [92]. (**B**) Silk fibroin with additives of ethylene glycol and triethylene glycol to release insulin over 4 days [87]. Reproduced with permissions: (**A**) is from John Wiley and Sons, 2017; (**B**) is from American Chemical Society, 2020.

Wound sites often can be repaired with differentiated cells or stem cells to aid in the regeneration process. Mesenchymal cells are stem cells that are often used in tissue regeneration due to their differentiating capacity. The problem with using mesenchymal stem cells is that they are not able to survive for prolonged periods in the wound site. An example of a biocompatible self-healing biopolymer hydrogel carrier for mesenchymal cells is a mix of carboxymethyl chitosan and carboxymethyl cellulose dialdehyde to create a gelatin cell carrier [86]. This specific hydrogel has sheer-thinning abilities and self-healing capabilities for the survival of the mesenchymal cells and allowing stem cell paracrine signaling for proper wound repair increase in cell proliferation, as seen in Figure 5C [76,86]. Another hydrogel used is a chitin nanofiber (CNF) hydrogel which encapsulated bone marrow mesenchymal stem cells (BMSC) for wound healing. This hydrogel presented the ability to promote the bone marrow stem cells differentiation into different and beneficial cell by protecting the cells to keep them viable for wound repair as seen in Figure 6A [92]. As seen with the BMSC hydrogel, the subject was able to heal the wound much more quickly compared to the other treatment option, showing the immense potential that hydrogels carry moving forward. It is believed that in the future, treatments like these will become much cheaper to produce and administer and because of their promising results we should see better outcomes when it comes to deeper wounds with less scar tissue and damage done to the overall area.

Silk hydrogels were also studied for their mechanics and properties for neural injury repair, as fibronectin- and NT-3-functionalized silk gels elicit increased axonal bundling suggesting their use in bridging nerve injuries. These results support silk hydrogels as soft and sustainable biomaterials for neural tissue engineering [93]. Therefore, biopolymer-based hydrogels can be incredibly advantageous for neural repairs. A different hydrogel was gelatin/microbial transglutaminase (mTG) hydrogel encapsulating human adipose-derived stem cell spheroids (hASC) to repair wounds. This hydrogel facilitates a good environment for the hASC spheroids to grow demonstrating a higher efficacy in making the wound site smaller [94]. These stem cells delivered via hydrogels demonstrate potential for allowing quicker and more effective methods of wound healing without being invasive to the body.

### 5.2. Drug and Biomolecule Delivery

Biopolymer-based hydrogels have also recently been used as controlled release methods to deliver drugs to the intended location by protecting against hostile environments within the body (Table 2). These hydrogels are often loaded with a drug that can be released when triggered by specific triggers such as pH, temperature, or chemical ligands. Often, hydrogels are synthesized so that they are biocompatible, biodegradable, and allow the drug to diffuse through the hydrogel based on the diffusion coefficient of the drug through the hydrogel network [8]. Some major examples of hydrogel drugs and biomolecule deliveries are the delivery of cancer therapy and the delivery of insulin particles to lower blood sugar levels [76].

#### 5.2.1. Cancer Therapy

Hydrogels can be used to help give localized controlled release of cancer drugs to desired locations without the adverse off-target effects. Thermo-responsive drug delivery hydrogel systems have been seen to work well as they change states at different temperatures to help with the loading and release of cancer drugs [95]. An example of the thermo-responsive hydrogel was chitosan/Disulfiram (DSF)-loaded hydrogel which released the anticancer drug DSF. This hydrogel system demonstrated higher cellular uptake than the cellular uptake of the DSF drug alone while having a sustained delivery to help with cancer therapy [96]. Another example of a hydrogel system was a chitosan-base hydrogel neutralized with beta-glycerophosphate which delivered paclitaxel anti-cancer drug within the body. This hydrogel with its localized delivery ability demonstrated sustained by only releasing 32% of the drug loaded into the hydrogel at day 17 with noticeable inhibition of tumor growth [97].

Another hydrogel composed from gelatin seeded with adipose-derived stem cells were studied to see if they could mimic the physical and chemical properties of natural ECM of the adipose cells and the tissue they compose. It was seen to be a good option moving forward for breast cancer treatment as this hydrogel was sustainable for around 21 days in vitro and this scaffold had similar mechanical properties to breast tissue [98]. A different hydrogel system was a mucin glycoprotein-based hydrogel which was loaded with hydrophobic drug paclitaxel and a hydrophilic drug polymyxin B to prevent bacteria. This hydrogel system was also reported to sustainably release both drugs for more than four weeks allowing for reduced viability of HeLA epithelial cancer cells and the prevention of bacterial growth as seen in Figure 5E [88]. These hydrogel drug delivery systems aid in releasing drugs in the desired location allowing for higher efficacy of the drug and lower side effects as the drug becomes more localized preventing harm to functioning and healthy organs. However, due to the nature of cancer and the fact that patients sometimes need harsh treatments to destroy the cancer cells, progress in this area is not going as fast as scientists in the field would like. Future studies can be done with cancer subjects to see if hydrogels are able to provide better results that are expected.

#### 5.2.2. Insulin Drug Delivery

Recently, it has been noticed that the current delivery methods for insulin are not very efficient. Patients are required to constantly check their glucose levels and must inject themselves with insulin on multiple occasions to maintain blood glucose levels. A new method of using injectable SF (silk-fibroin) protein hydrogel (iSFH) has been formulated to have a more efficient method of controlled release of insulin. This iSFH hydrogel has been found to control insulin release and maintain blood glucose levels for around 5 days as seen in Figure 5D and Figure 6B [87]. Another insulin delivery system of alginate-gum tragacanth has a polyelectrolyte complexion with chitosan and was able to retain almost all the insulin entrapped when going down areas with pH similar to our gastric. For this hydrogel, it was found that the hydrogel was able to release the insulin in a sustained fashion in pH like that in the intestinal tract demonstrating its ability to respond to different pH environments for localized insulin delivery [17]. A different insulin delivery system of polysaccharide-based Salecan hydrogels were able to carry insulin and provide a controlled release due to environmental pH. For this hydrogel, it was found that at acidic pH the insulin was not secreted but at neutral pH, the insulin was released demonstrating insulin reaching desired locations in the body [99]. These biopolymer-based hydrogel systems present more efficient methods of controlling blood glucose levels for those who are diabetic without having to constantly inject insulin or worry whether all the injected insulin reaches the desired location.

## 6. Conclusions

Hydrogels are unique polymeric materials that contain hydrophilic chains providing the ability to retain a large volume of water in their interstitial structures. Biopolymers especially can be used to construct hydrogels for specific applications and unique properties. An array of different biopolymers, such as saccharides and peptides, can be formulated into hydrogels, each with its advantages and intended applications. Examples of polypeptides that have been implemented as hydrogels include collagen, gelatin, and elastin. On the side of polysaccharide hydrogels, a few include alginate, cellulose, and glycosaminoglycans and even nucleic acids can be used to construct unique hydrogels. There have already been incredibly creative ways of combining and layering different hydrogels, such as combining two different hydrogels that are affected by different stimuli and using them to create a tubular structure to treat damaged blood vessels in the body. Double-network hydrogels with unique mechanical properties were also invented because of the different network densities in the hydrogel. They would be beneficial for ligament treatments as the outer network can resist the shear forces, and the inner network is able to resist the tensional forces.

Many theories are used to predict the structure of hydrogels and analyze the gels. By combining these derived theories with computational methods, one can even predict the structure and functionality of hydrogels before synthesis, which will lead to faster development of hydrogel uses for biomedical purposes. With the different types of gels that are present (pH-sensitive, heat-sensitive, electro-sensitive, light-sensitive and shape-changing), there are endless applications and creative uses for the development of novel hydrogels, the efficiency at which we can test, and the material design that we can improve to unprecedented levels.

Hydrogels can be created in several different ways, from standard fabrication methods using basic chemical reactions, to innovative 3D printing designs. These manufacturing wonders are only the start of hydrogel production and are paving the way to great production advancements. Hydrogels are viable options for tissue regeneration via encapsulated cells in hydrogels aimed for desired tissue locations. Drug and molecule releasing hydrogels such as insulin-releasing hydrogels and cancer drug-releasing hydrogels can deliver insulin and cancer drugs, respectively, to their desired locations to maximize results and to minimize harmful drug effects. These successful applications of hydrogels mainly made since 2015 in the medical landscape have presented further avenues to synthesize hydrogels that encapsulate other drugs, cells, and molecules. These newer hydrogels will allow for less invasion tissue regeneration, possibly avoiding surgical procedures entirely, and more effective drug delivery for better patient outcomes. For example, with back pain issues plaguing the world, new hydrogels could be used to alleviate the pain that people experience in their lives, without the need for invasive surgeries.

In the future, development of more refined biopolymer-based hydrogels such as mixtures of natural polymers with different properties like elastin-cellulose and elastin-alginate for tissue regeneration would be beneficial for tissue engineering purposes. Ultimately, as hydrogels have major potential for tissue regeneration and drug delivery, it will be an interesting endeavor for other researchers to develop new and innovative biopolymer-based hydrogels that are able to have longer and more sustained controlled release of drug and degrade less over time for higher rates of tissue regeneration. Therefore, it is believed that hydrogel therapy will dominate future medical treatments due to its versatility, biocompatibility, and tunability.

## Figures and Tables

**Table 1:** Different types of hydrogels based on the stimulus that they respond to, and the potential applications that they have in the biomedical field.

Type of Gel	Stimuli	Biomedical Applications
pH-sensitive	Acid/base environments	Drug delivery, cell delivery [62,63]
Thermo-sensitive	Heating/cooling, possible EM waves such as IR	Wound dressing, drug delivery, tissue engineering, imaging techniques [64,65]
Electro-sensitive	Magnetic field or electric stimulus	Controllable drug release, artificial muscles, controllable valves [66,67,68]
Light-sensitive	EM Waves	Regulation of stimulating factors, biological materials, and biosensors [69]
Shape-changing	Solvent environments	Drug delivery, cell delivery [13]

**Table 2:** Overview of biopolymer materials used in hydrogel applications.

Material	Biopolymer Category	Fabrication Method	Recent Application (s)
Collagen	Polypeptide	Demineralization	Drug delivery, tissue engineering, implants [24]
Gelatin	Polypeptide	Boiling natural products	Drug delivery, tissue engineering [29]
Elastin	Polypeptide	Polymerization of tropoelastin monomers	Tissue engineering [32]
Alginate	Polysaccharide	Extraction from cell walls of algae	Cell delivery [39]
Cellulose	Polysaccharide	Extrusion from dissolved natural materials like wood pulp	Biomedical devices, tissue engineering, drug delivery [46]
Glycosaminoglycans	Polysaccharide	Synthesis of UDP derived activated sugars	Tissue engineering [80]
DNA	Nucleic Acid	Light-directed combinational chemical synthesis	Drug delivery, tissue engineering, cancer therapeutics, biosensing [51]
